# Evolving Face Mask Guidance During a Pandemic and Potential Harm to Public Perception: Infodemiology Study of Sentiment and Emotion on Twitter

**DOI:** 10.2196/40706

**Published:** 2023-02-27

**Authors:** Divya Ramjee, Catherine C Pollack, Marie-Laure Charpignon, Shagun Gupta, Jessica Malaty Rivera, Ghinwa El Hayek, Adam G Dunn, Angel N Desai, Maimuna S Majumder

**Affiliations:** 1 School of Public Affairs American University Washington, DC United States; 2 Geisel School of Medicine Dartmouth College Lebanon, NH United States; 3 Institute for Data, Systems, and Society Massachusetts Institute of Technology Cambridge, MA United States; 4 Comp Epi Dispersed Volunteer Research Network Boston, MA United States; 5 Boston Children’s Hospital Boston, MA United States; 6 Harvard Medical School Boston, MA United States; 7 School of Medical Sciences The University of Sydney Sydney Australia; 8 Department of Internal Medicine, Division of Infectious Diseases University of California-Davis Health Medical Center Sacramento, CA United States

**Keywords:** face masks, COVID-19, Twitter, science communication, political communication, public policy, public health, sentiment analysis, emotion analysis, infodemiology, infoveillance

## Abstract

**Background:**

Throughout the COVID-19 pandemic, US Centers for Disease Control and Prevention policies on face mask use fluctuated. Understanding how public health communications evolve around key policy decisions may inform future decisions on preventative measures by aiding the design of communication strategies (eg, wording, timing, and channel) that ensure rapid dissemination and maximize both widespread adoption and sustained adherence.

**Objective:**

We aimed to assess how sentiment on masks evolved surrounding 2 changes to mask guidelines: (1) the recommendation for mask use on April 3, 2020, and (2) the relaxation of mask use on May 13, 2021.

**Methods:**

We applied an interrupted time series method to US Twitter data surrounding each guideline change. Outcomes were changes in the (1) proportion of positive, negative, and neutral tweets and (2) number of words within a tweet tagged with a given emotion (eg, trust). Results were compared to COVID-19 Twitter data without mask keywords for the same period.

**Results:**

There were fewer neutral mask-related tweets in 2020 (β=–3.94 percentage points, 95% CI –4.68 to –3.21; *P*<.001) and 2021 (β=–8.74, 95% CI –9.31 to –8.17; *P*<.001). Following the April 3 recommendation (β=.51, 95% CI .43-.59; *P*<.001) and May 13 relaxation (β=3.43, 95% CI 1.61-5.26; *P*<.001), the percent of negative mask-related tweets increased. The quantity of trust-related terms decreased following the policy change on April 3 (β=–.004, 95% CI –.004 to –.003; *P*<.001) and May 13 (β=–.001, 95% CI –.002 to 0; *P*=.008).

**Conclusions:**

The US Twitter population responded negatively and with less trust following guideline shifts related to masking, regardless of whether the guidelines recommended or relaxed mask usage. Federal agencies should ensure that changes in public health recommendations are communicated concisely and rapidly.

## Introduction

### Background

Public health recommendations rapidly evolve when contending with a fast-developing pandemic like COVID-19, and optimized communication is critical to positively impact health-related behaviors and outcomes. Effective communication of trustworthy information has proven key to overcoming public health crises in the past, particularly when the coordinated effort of entire populations has been required [1]. During global health crises, public institutions are considered trusted sources of information, but they face challenges in providing evidence-based guidance on real-time preventative measures [1,2]. The Centers for Disease Control and Prevention (CDC) is one of the leading federal agencies in the United States charged with protecting public health. It provides primary directives for public health measures that are disseminated to the general public via various outlets, including social media platforms [3-6].

### Public Health Communication

Messaging strategies are a key tenet of strategic communication. Public health communication in particular is driven by an ecological foundation, recognizing that public health is affected by social, behavioral, political, and environmental factors [7]. As such, it requires multilevel strategies for disseminating information, including “tailored messages at the individual level, targeted messages at the group level, social marketing at the community level, media advocacy at the policy level, and media campaigns at the population level” [7]. In 1993, the director of the CDC established that health communication should be considered an integral component of their prevention programs and created a 10-step messaging framework to promote changes in awareness, attitudes, and beliefs that may ultimately influence health behaviors [8]. This framework has evolved over time, notably with the addition of the crisis and emergency risk communication (CERC) considerations in the aftermath of 9/11 and subsequent anthrax attacks [9].

The CERC strategy generally follows a 5-phase paradigm: (1) the pre-crisis phase, involving potential response preparedness; (2) the initial phase, when the outbreak begins and information is often fluid and possibly confusing; (3) the maintenance phase, involving clarifying information on risk perceptions and correcting misinformation; (4) the resolution phase, when the outbreak is resolved; and (5) the evaluation phase, involving review of lessons learned [9-11]. Over the past decade, public health organizations have struggled to adequately address public concerns during outbreaks of Ebola, H5N1 avian influenza, and Zika, and these organizations have encountered similar obstacles during the first 3 phases of the COVID-19 pandemic [12-15]. This is especially apparent when countering misinformation regarding individual-level behaviors [16-18].

### Sentiment and Emotion

Growing research demonstrates the association between trust in government and public health organizations and their effectiveness in communicating public health information for optimal individual-level compliance [12,19-22]. According to a 2015 poll, only 19% of Americans trusted the US federal government always or most of the time, while 71% of Americans expressed trust in the CDC in 2017 [3,23]. However, in 2022, trust in the CDC fell to 50% [24]. Considering the stature of the CDC in society, its communications—especially those on social media, where they may get the most amount of attention by the general population—play an essential role in preparedness and response efforts during all phases of disease outbreaks.

Health communication generally relies on adapting established theories and models of behavior for each public health campaign. These include the theory of reasoned action, health belief model, social learning/cognitive theory, extended parallel process model, diffusion of innovation, and social marketing [25,26]. However, these decision-making theories do not effectively consider the influence of attitudes, emotions, and cultural norms on ultimate behaviors, as suggested by an assessment of HIV/AIDS communication campaigns for prevention [26]. Additionally, disseminating evolving and corrective information throughout a communication campaign can also present challenges. As people receive newer information, interpretation of this updated information follows an attitude-consistent manner despite a willingness to accept the information as factual, wherein individuals are more willing to express distrust in the credibility of the information [27]. Analysis of the CDC’s communication campaign during the Zika epidemic suggested that updated or corrective information positively impacted public health perceptions in the initial months of the epidemic and did not affect the credibility of the CDC [28,29]. However, more recent research from the current COVID-19 pandemic has indicated that the positive effects of updates to information may be short-lived [30].

### The Role of Twitter

During health crises, Twitter has proven effective at identifying public concerns over the health consequences of emerging disease outbreaks and tracking disease activity based on users’ health behavior [31-36]. Prior studies have demonstrated that Twitter data can be used to understand public sentiment in real time and tailor individualized public health messages based on user interest and emotion [34,37,38]. From February 2020 through May 2021, the CDC changed guidelines on face masks (herein referred to as masks) multiple times, from initially discouraging mask use at the beginning of the pandemic among non–health care workers, to recommending mask use for all individuals, to suggesting masks were optional for individuals who were vaccinated, to again recommending the use of masks for all individuals during another surge in case counts of COVID-19. During a February 2022 press call, CDC Director Rochelle Walensky cautioned that “None of us know what the future holds for us and for this virus.... And we need to be prepared and we need to be ready for whatever comes next. We want to give people a break from things like mask wearing when our levels are low, and then have the ability to reach for them again if things get worse in the future” [39]. Given this evolving messaging toward mask guidelines, it is critical to explore how mask-related decisions thus far during the pandemic have impacted public sentiment and emotion.

To evaluate how changes in CDC mask guidelines (ie, recommendation and relaxation) impacted social media discourse, this study applies methods from computational epidemiology and the social sciences to rapidly evaluate vast amounts of publicly accessible social media data [40]. In particular, this study focuses on Twitter, given its role in the proliferation of public health communications [41]. Our research highlights the complex issues of public health communication confronting federal agencies, particularly the CDC, and the public at large. Formally, our objective was to evaluate changes in public perception (as measured through sentiment and emotion expressed on Twitter) surrounding the April 3, 2020, and May 13, 2021, masking guidelines made by the CDC. We investigate the impacts of changing mask guidelines on public sentiment and emotions toward mask use and hypothesize that changing guidelines (1) influence public sentiment toward mask use but (2) do not change perceptions of the CDC’s credibility, specifically trustworthiness.

## Methods

### Data Collection

Tweets containing at least one COVID-19–related keyword were collected using repeated searches via version 1.1 of the official Twitter application programming interface (API). The API was queried in several steps as part of a separate project conducted by team members at the University of Sydney. Starting on February 10, 2020, the Search Tweets end point was run on an automated schedule every 7 days to collect tweets based on a specific set of COVID-19–related queries (Table 1) [42]. When running, the process would request 100 COVID-19–related tweets from the API, save those tweets to a database, and then request the next 100 tweets until it ran out of tweets to gather. The frequency of requests was 450 times per 15 minutes (due to rate limits imposed by the Twitter API), resulting in 45,000 tweets per 15 minutes. Starting on March 17, 2020, this process was switched to the Twitter Stream API, which had an ongoing open connection with Twitter [43]. In this new process, whenever a tweet matching the keywords of interest was posted by a user, it was sent to the database within seconds.

Analysis was restricted to original tweets (ie, retweets were omitted) in English from users based in the United States. The GeoNames geographical database was used to identify user location based on the account location field (ie, the location provided by a Twitter user in their public profile, if any). The data set was then restricted to only tweets that contained mask-related terminology, and these keywords were selected based on the collective expertise of the research team (Table 1). The comparator data set was generated by first extracting tweets that contained at least one COVID-19 keyword but no mask keywords. A random number of comparator tweets were then selected for each day such that the number of comparator tweets for any given day was equivalent to the number of mask tweets on that day. For example, if there were 500 mask tweets on March 2, 500 random comparator tweets that contained COVID-19 terminology but no mask terminology would be selected for that day. The daily number of tweets used for analysis in 2020 and 2021 are provided in Multimedia Appendix 1, Table S1, and Multimedia Appendix 2, Table S2, respectively.

Data were evaluated during 2 time periods: March 1, 2020, to June 30, 2020, and April 1, 2021, to June 13, 2021. During the first time period, on April 3, 2020, the CDC set new guidelines that cloth or fabric face coverings (eg, masks) be used as an additional and voluntary preventive measure that could protect others from COVID-19 transmission [44-46]. This was a reversal of guidelines made during a tweet on February 27, 2020, which stated that the CDC did “not currently recommend the use of masks to help prevent novel coronavirus,” instead encouraging their Twitter followers (4.7 million on the main @CDCGov account as of April 5, 2022) to stay at home when sick and wash hands with soap and water to slow the spread of disease [47]. Amid a shortage of personal protective equipment, CDC officials reasoned that this position might reduce the likelihood of stockpiling by the general public and save hospital-grade masks for health care workers [48].

The second time period of analysis (ie, April 1, 2021, through June 13, 2021) was chosen based on a revision to the guidelines by the CDC, which noted on Twitter, “[i]f you are fully vaccinated against #COVID19, you can resume activities without wearing a mask.” Two months later, this recommendation was revoked amid a surge of the SARS-CoV-2 Delta variant [49].

**Table 1:** Search keywords used to collect and filter tweets for inclusion in the data set.

Category	Keywords
Coronavirus	covid, covid-19, covid19, ncov, sars-cov-2, sarscov2, 武汉肺炎, 武汉疫情, ncov2019, 2019ncov, ncov19, 19ncov, coronoravirus, wuhan virus, covid_19, coronavirususa, coronovirus, coronavid19, coronavirusupdate, coronaoutbreak, coronavirusaustralia, coronvirus, coronaalert, covid—19
Face mask (substring search)	mask, n95, cloth face, cloth cover, face cover, mouth cover, nose cover, cover your face, coveryourface

### Sentiment Analysis and Emotion Analysis

Links, hashtag symbols, and @ mentions were removed from tweets prior to calculating sentiment scores using the Valence Aware Dictionary and Sentiment Reasoner (VADER). This methodology, which was specifically designed for social media data, incorporates emojis, punctuation, capitalization, and negation when calculating the compound sentiment score (ranging from –1 to 1). Tweets with a score above 0.05 were labeled as “positive” and those below –0.05 were labeled as “negative”; all other tweets were labeled as “neutral” [50].

Each tweet was also mapped to a set of emotions based on the National Research Council of Canada (NRC) Word Emotion Lexicon [51]. The NRC associates each word with at least 1 of 8 emotions—anger, anticipation, disgust, fear, joy, sadness, surprise, and trust—on a scale from 0 to 1. Before calculating emotionality, all HTML escape characters, stop words, punctuation, and numbers were removed, followed by conversion to lower case and tokenization. For a given tweet, the final score corresponding to each emotion was calculated by summing emotion scores across tokens corresponding to that emotion.

### Statistical Analysis

An interrupted time series analysis was used to evaluate the change in sentiment and emotion outcomes around the 2 shifts in guidelines. Each model contained a term for the pre-event trend (ie, recommendation for mask use or relaxation of this recommendation), an instantaneous effect on the day of the event, and a postevent trend. For each year, the outcomes of interest included change in average daily compound sentiment score, percent of tweets with a given sentiment (ie, positive, negative, or neutral, with individual models for each sentiment), and total emotion score (ie, the sum of words tagged with a given emotion of interest, with individual models for each emotion). For all outcomes, models were evaluated individually and relative to the comparator data set. Analysis was conducted in R (version 4.1.2; R Foundation for Statistical Computing) using the RStudio Integrated Development Environment (version 2021.09.0). A *P* value of less than .05 (*P*<.05) was considered statistically significant, and the authors determined there were not enough statistical comparisons to warrant additional hypothesis correction methods. This was due to the exploratory nature of this study and the decision that type II errors (eg, failing to identify a true association) were more deleterious than type I errors (eg, identifying a spurious association) [52].

## Results

### April 3, 2020, CDC Mask Recommendation Guideline

**Figure 1:**
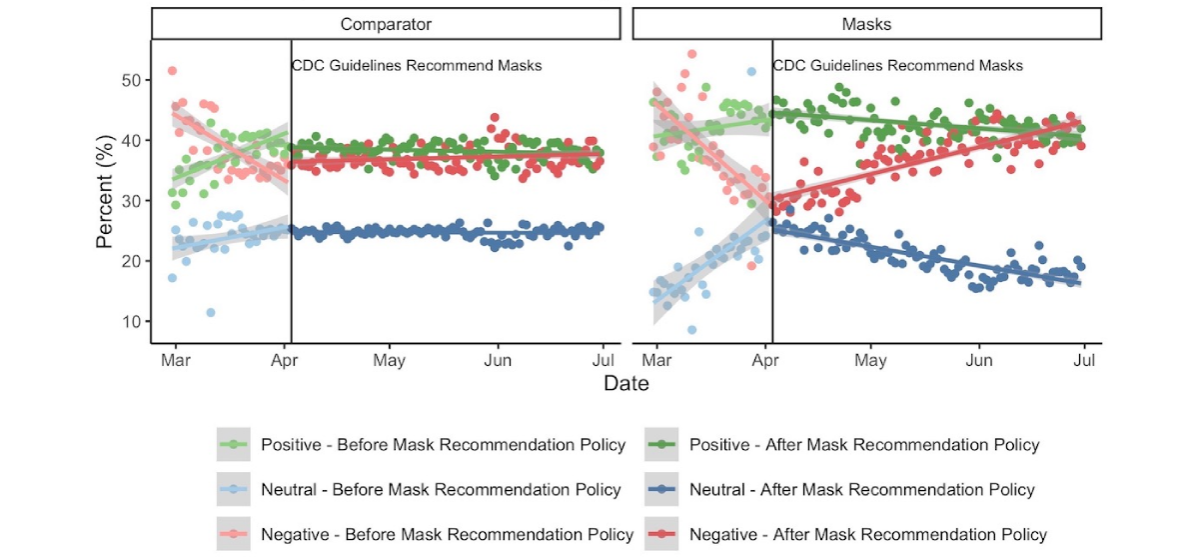
Daily change in the distribution of positive, negative, and neutral tweets among mask and nonmask COVID-19 tweets between March 1 and June 30, 2020. CDC: US Centers for Disease Control and Prevention.

There were 1,106,756 mask-related tweets during the 4-month period surrounding the first guideline (ie, the CDC mask recommendation) with an equivalent quantity collected for the comparator. Between February 29, 2020, and June 30, 2020, mask-related tweets were more positive than comparator COVID-19 tweets (β=.06, 95% CI .05-.07; *P*<.001; Figure 1). In particular, the percent of positive tweets on any given day was 4.43 percentage points higher than concurrently observed in the comparator (95% CI 3.82-5.03; *P*<.001), while the percent of neutral tweets was lower (β=–3.94, 95% CI –4.68 to –3.21; *P*<.001). After the mask recommendation on April 3, 2020, the proportion of negative tweets within the mask-related data set increased (β=.51, 95% CI .43-.59; *P*<.001). However, the average number of negative tweets on any given day was not substantially different from the comparator (β=–.49, 95% CI –1.31 to .33; *P*=.24; Figure 1).

In terms of emotion, mask-related tweets expressed an increasing level of trust (β=.004, 95% CI .003-.004; *P*<.001) but decreasing levels of both sadness (β=–.003, 95% CI –.004 to –.002 *P*<.001) and surprise (β=–.001, 95% CI –.001 to 0; *P*=.005) during the period preceding the April 3, 2020, CDC recommendation. However, the levels of sadness (β=.004, 95% CI .003-.005; *P*<.001) and surprise (β=.001, 95% CI 0-.001; *P*=.003) expressed in mask-related tweets increased following the CDC recommendation, while trust decreased (β=–.004, 95% CI –.004 to –.003; *P*<.001). The levels of anger, anticipation, disgust, or joy expressed on any given day did not substantially differ between the mask-related data set and the comparator. However, mask-related tweets expressed a higher level of trust (β=.131, 95% CI .122-.140; *P*<.001), but less sadness (β=–.042, 95% CI –.053 to –.031; *P*<.001) and surprise (β=–.026, 95% CI –.03 to –.021; *P*<.001) relative to the comparator data set.

### May 13, 2021, CDC Mask Relaxation Guideline

**Figure 2:**
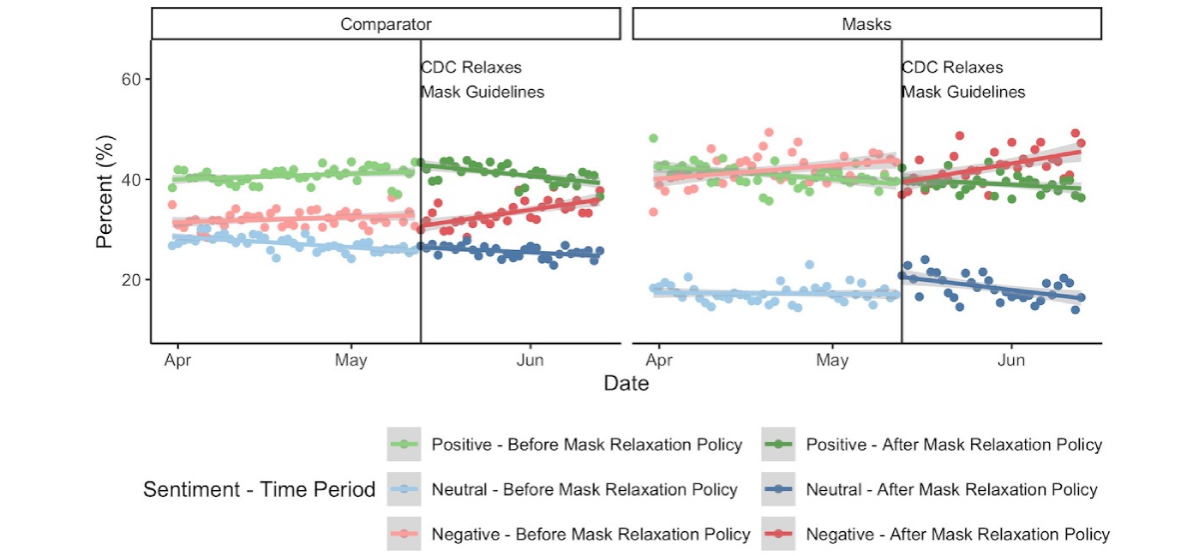
Daily change in the distribution of positive, negative, and neutral tweets within mask and non-mask COVID-19 tweets between April 1 and June 13, 2021. CDC: US Centers for Disease Control and Prevention.

There were 321,119 mask-related tweets during the 10-week period surrounding the second guideline shift (ie, the CDC mask relaxation), with an equivalent amount in the comparator. On any given day between April 1, 2021, and June 13, 2021, sentiment expressed in mask-related tweets was more negative than the comparator (β=–.06, 95% CI –.05 to –.06; *P*<.001; Figure 2). In particular, the proportion of negative tweets within the mask-related data set was 9.50 percentage points higher on average than in the comparator (95% CI 8.74-10.3; *P*<.001). During the same time period, the proportion of neutral tweets was 8.74 percentage points lower (95% CI –9.31 to –8.17; *P*<.001), and the proportion of positive tweets was 0.76 percentage points lower (95% CI –1.37 to –0.15; *P*=.02). Immediately after the mask relaxation on May 13, the proportion of negative tweets increased (β=3.43, 95% CI 1.61-5.26; *P*<.001), whereas the percent of neutral tweets decreased (β=–4.46, 95% CI –7.07 to –1.84; *P*=.001)

On any given day, and in all categories except the emotion of surprise (β=–.004, 95% CI –.009 to .001; *P*=.09), mask-related tweets expressed higher levels of emotion than tweets in the comparator. Before the mask recommendation was revoked, the levels of anger (β=.001, 95% CI 0-.001; *P*=.007), fear (β=.001, 95% CI .001-.002; *P*<.001), sadness (β=.001, 95% CI 0-.002; *P*=.001), and trust (β=.001, 95% CI 0-.001; *P*<.001) expressed in mask-related tweets increased daily. Following the mask recommendation relaxation, the level of anger continued to increase (β=.001, 95% CI 0-.002; *P*=.02), whereas trust decreased (β=–.001, 95% CI –.002 to 0; *P*=.008).

## Discussion

### Principal Findings

This study is among the first to characterize the evolution of mask-related content on Twitter surrounding the recommendation and relaxation of mask guidelines by the CDC during the COVID-19 pandemic. In summary, our study found that after both the 2020 mask recommendation and the 2021 mask relaxation a pronounced decrease in neutral tweets occurred. Following the 2020 mask recommendation, sentiment expressed in mask-related tweets was substantially more positive than in other COVID-19 tweets. In contrast, sentiment expressed in mask-related tweets following the 2021 mask relaxation was more negative. Furthermore, both mask-related data sets suggested higher levels of emotions than other COVID-19 tweets. In particular, both time periods were marked by a higher proportion of tweets expressing disgust before the change in guidelines and lower proportion of tweets expressing trust following the change. Our main findings suggest that shifts in guidelines emanating from the CDC may have a tangible, negative impact on the perception of mask use among United States–based Twitter users, with implications for the design of mask-wearing policies and other similar preventative health measures in the future.

Masks are a crucial public health tool to fight the spread of infections such as SARS-CoV-2. High adherence to mask-wearing policies may help reduce transmission during severe disease outbreaks, including pandemics [35]. However, mask use in the United States has become increasingly politicized and polarizing. Recent work evaluating the state of mask-related discourse on Twitter found that corresponding tweets expressed increasingly negative sentiment between March and July 2020, although that research did not focus on CDC announcements as interventions or include an extended time period after the relaxation [53]. Other research suggests that anti-mask rhetoric accounted for 10% of mask-related content between January and October 2020, with varying volume around key US guideline shifts [54]. These results corroborate our findings, namely that the mask-related discourse on Twitter was increasingly more polarized after the CDC announced the mask recommendation on April 3, 2020.

As online information-seeking behaviors increase, so do access and exposure to conflicting information and political infighting [55]. False information quickly and easily spreads via online social networks and, in tandem with fluctuating and confusing messaging during the initial phase of a public health emergency, promotes negative public sentiments and difficulties in preserving public trust [56-58]. Recent research indicates that efforts to disseminate corrective information during the maintenance phase of a public health crisis are ineffective at both countering misconceptions and gaining support for the adoption of preventive health-related behaviors [13]. This finding suggests that, despite the quickly changing atmosphere, concise and consistent messaging is critical in the precrisis and initial phases of a public health emergency for highest individual-level adherence to preparedness and prevention measures. While the CDC attempted to provide clear messaging regarding mask use, its response was perceived as slow relative to the speed at which clinical findings were released. Furthermore, this perceived slow response, coupled with positions that conflicted with other global health organizations, such as the World Health Organization, may have inadvertently contributed to feelings of confusion and mistrust among the general public [59,60]. This effect may have been captured within our data set as the decreased levels of trust-related terminology expressed within tweets following each shift in guidelines. Furthermore, the fact that mask tweets within our data were substantially more negative than the comparator in 2021 may suggest a high degree of preexisting mask fatigue, and the subsequent additional increase in negative tweets following the relaxation recommendation on May 13 may indicate discontent at the lack of transparency from the CDC.

### Health Communications Recommendations

Although Twitter and other social media platforms can be leveraged to rapidly inform the public of important recommendations, this study suggests that there may be negative consequences for public support when such messages are not communicated effectively. In our study, this is illustrated by the decrease in levels of trust expressed by United States–based Twitter users following both guideline shifts in 2020 and 2021 [61]. Based on these findings, we believe that there are several communication strategies that should be considered during future health emergencies to ensure that the general public maintains trust in government agencies.

First, it is imperative that a consistent message is embraced by diverse, respected professionals in the field. Along with trusted government agencies like the CDC, this may also include public health and medical experts, research scientists, politicians, science communications specialists, and even popular influencers and celebrities in order to reach multiple demographics [62]. This message should be authentic and transparent about the fact that information will likely evolve, especially during ongoing crises. Second, it is important for government agencies to monitor social media engagement and promote dialogue to understand perceptions and motives for health practice. Each social media platform reaches a different target audience, so multiple accounts across platforms may be warranted to ensure that as many individual opinions are considered as possible. While social media is not generalizable to the entire population, it can help supplement traditional epidemiologic measures of data collection, such as representative surveys, that may be more reliable but are more costly to coordinate. Third, it may be salient for government agencies to develop educational materials that directly address and correct incorrect perceptions, attitudes, and behaviors. These materials must be “living” documents that are continuously updated as new misperceptions emerge. They should also be made widely accessible and promoted through multiple media outlets, including social media. Taken together, the increased transparency and access afforded by consistent messaging, increased social media engagement, and easily understood education materials could help ensure that the general public continues to look to government agencies for guidance during future health emergencies, especially those that are tumultuous.

### Limitations and Future Directions

Our study is the first to evaluate the sentiment and emotion of mask-related tweets in the United States surrounding 2 key guideline shifts made by the CDC relative to a matched comparator data set of other COVID-19 tweets during the same period. However, there are several limitations to note. First, the reliance on keywords to collect relevant tweets may introduce some selection bias. Specifically, filtering tweets with keywords may exclude tweets that discuss the topic of interest but contain a misspelling. Additionally, some tweets, such as automated advertisements, may contain the appropriate keywords but are not relevant to public opinion. Given the persuasive nature of advertising, it is likely that their inadvertent inclusion might have biased our results and skewed the estimation of positive sentiment to be higher than that which was present in the general public. Future work could use the *–is:nullcast* filter, which was not available in the version of the Twitter API that was used to collect the data for this study (version 1.1), to ensure that these tweets were removed. Second, tweets were restricted to those posted by users located within the United States based on the geotag in the user profile. However, users reporting location information in their profile may be different from those without such content. Future work should attempt to identify and leverage other methods to assess where Twitter users are located. Third, sociodemographic data were not available, which may impact generalizability. While social media studies can provide rapid insights during health emergencies, they are not necessarily representative of the overall US population; specifically, Twitter users tend to be younger, more educated, and have a higher average income than the general US population [63]. Fourth, findings are based on aggregate analysis at the national level, and future work could characterize patterns at a state level. Lastly, future work could employ alternative natural language processing and sentiment analysis methods, such as emoji analysis or word embeddings, to understand how results may change.

### Conclusions

Our study supports findings from prior research on the importance of formulating clear public health communications and disseminating accurate public health guidance on social media. Specifically, we found that tweets surrounding the 2020 mask recommendation and 2021 mask relaxation were more polarizing and contained less trust-related terminology than those before the guidelines were announced. Furthermore, while mask-related tweets posted in 2020 were more positive than other COVID-19 tweets, mask-related tweets in 2021 were more negative. The change in sentiment observed in 2021 may signal frustration among Twitter users about public health discourse centered around masks and recognition that the initial mask relaxation change may have been premature.

Gaining insight into how the general public engages on social media platforms, perceives preventative public health measures imposed during the COVID-19 pandemic, and reacts to shifts in guidelines declared by the US government is of utmost importance for policy makers, health workers, and interested stakeholders. Official communications that include concise information backed by systematic data are critical to ensure widespread adoption and sustained adherence to public health interventions. However, the rapid spread of COVID-19 and the evolving evidence around its mitigation led to confusion from the public surrounding the fluctuating mask guidelines. When messaging remains unclear and lacks direction, public sentiment and trust in authoritative entities erode. This is especially true for masks, where policy recommendations pertaining to mask use constantly shifted throughout 2020 and 2021, sometimes without clear evidence presented to the public [59,60]. Given that health officials have noted that mask guidelines may serve as a recurring tool to mitigate contagion spread during peak infection (both in the current pandemic and in response to future pandemic threats or emerging biothreats) it is imperative that institutions such as the CDC use consistent, clear communication strategies that align with other major health organizations and the broader scientific community. This will ensure that the potential for polarization is minimized while trust in the government and adherence to preventive measures is maximized.

## Appendix

Multimedia Appendix 1 Supplementary Table S1: Number of tweets for a given day between March 1, 2020, to June 30, 2020.

Multimedia Appendix 2 Supplementary Table S2: Number of tweets for a given day between April 1, 2021, to June 13, 2021.

## Abbreviations

API: application programming interface
CDC: US Centers for Disease Control and Prevention
CERC: crisis and emergency risk communication
NRC: National Research Council of Canada
