# Supplementary material for: Evolving Face Mask Guidance During a Pandemic and Potential Harm to Public Perception: Infodemiology Study of Sentiment and Emotion on Twitter
Source: J Med Internet Res. 2023 Feb 27;25:e40706. doi: 10.2196/40706 (PMC9973548; doi:10.2196/40706)
Supplement: Multimedia Appendix 1 [file jmir_v25i1e40706_app1.docx]

**Table S1.** Number of tweets for a given day between March 1, 2020, to June 30, 2020

| Date | Number of Tweets |
| --- | --- |
| 02-29-2020 | 108 |
| 03-01-2020 | 373 |
| 03-02-2020 | 489 |
| 03-03-2020 | 480 |
| 03-04-2020 | 525 |
| 03-05-2020 | 463 |
| 03-06-2020 | 377 |
| 03-07-2020 | 379 |
| 03-08-2020 | 322 |
| 03-09-2020 | 443 |
| 03-10-2020 | 290 |
| 03-11-2020 | 35 |
| 03-12-2020 | 334 |
| 03-13-2020 | 1386 |
| 03-14-2020 | 343 |
| 03-15-2020 | 449 |
| 03-16-2020 | 1320 |
| 03-17-2020 | 1525 |
| 03-18-2020 | 1445 |
| 03-19-2020 | 1912 |
| 03-20-2020 | 4703 |
| 03-21-2020 | 5971 |
| 03-22-2020 | 4244 |
| 03-23-2020 | 6126 |
| 03-24-2020 | 1581 |
| 03-25-2020 | 5792 |
| 03-26-2020 | 6079 |
| 03-27-2020 | 5877 |
| 03-28-2020 | 10922 |
| 03-29-2020 | 8434 |
| 03-30-2020 | 9928 |
| 03-31-2020 | 10930 |
| 04-01-2020 | 9932 |
| 04-02-2020 | 11133 |
| 04-03-2020 | 17492 |
| 04-04-2020 | 14835 |
| 04-05-2020 | 10966 |
| 04-06-2020 | 11979 |
| 04-07-2020 | 11515 |
| 04-08-2020 | 10861 |
| 04-09-2020 | 9207 |
| 04-10-2020 | 9827 |
| 04-11-2020 | 6856 |
| 04-12-2020 | 5810 |
| 04-13-2020 | 7545 |
| 04-14-2020 | 8165 |
| 04-15-2020 | 9673 |
| 04-16-2020 | 9545 |
| 04-17-2020 | 9979 |
| 04-18-2020 | 8417 |
| 04-19-2020 | 7027 |
| 04-20-2020 | 8335 |
| 04-21-2020 | 8304 |
| 04-22-2020 | 8377 |
| 04-23-2020 | 8863 |
| 04-24-2020 | 7491 |
| 04-25-2020 | 6053 |
| 04-26-2020 | 5288 |
| 04-27-2020 | 7295 |
| 04-28-2020 | 15207 |
| 04-29-2020 | 11467 |
| 04-30-2020 | 10248 |
| 05-01-2020 | 8720 |
| 05-02-2020 | 6866 |
| 05-03-2020 | 7442 |
| 05-04-2020 | 9687 |
| 05-05-2020 | 10773 |
| 05-06-2020 | 9859 |
| 05-07-2020 | 10347 |
| 05-08-2020 | 11744 |
| 05-09-2020 | 9409 |
| 05-10-2020 | 7749 |
| 05-11-2020 | 11427 |
| 05-12-2020 | 9545 |
| 05-13-2020 | 9717 |
| 05-14-2020 | 11418 |
| 05-15-2020 | 9961 |
| 05-16-2020 | 7679 |
| 05-17-2020 | 8904 |
| 05-18-2020 | 10486 |
| 05-19-2020 | 10599 |
| 05-20-2020 | 11159 |
| 05-21-2020 | 11568 |
| 05-22-2020 | 9981 |
| 05-23-2020 | 9023 |
| 05-24-2020 | 8882 |
| 05-25-2020 | 10692 |
| 05-26-2020 | 11866 |
| 05-27-2020 | 11197 |
| 05-28-2020 | 11176 |
| 05-29-2020 | 10126 |
| 05-30-2020 | 6834 |
| 05-31-2020 | 6150 |
| 06-01-2020 | 6302 |
| 06-02-2020 | 6263 |
| 06-03-2020 | 6667 |
| 06-04-2020 | 6331 |
| 06-05-2020 | 8421 |
| 06-06-2020 | 8154 |
| 06-07-2020 | 5843 |
| 06-08-2020 | 7290 |
| 06-09-2020 | 7422 |
| 06-10-2020 | 9047 |
| 06-11-2020 | 10548 |
| 06-12-2020 | 11578 |
| 06-13-2020 | 9388 |
| 06-14-2020 | 9898 |
| 06-15-2020 | 13264 |
| 06-16-2020 | 14721 |
| 06-17-2020 | 17782 |
| 06-18-2020 | 20869 |
| 06-19-2020 | 19671 |
| 06-20-2020 | 17484 |
| 06-21-2020 | 9443 |
| 06-22-2020 | 15105 |
| 06-23-2020 | 17752 |
| 06-24-2020 | 23261 |
| 06-25-2020 | 26181 |
| 06-26-2020 | 28550 |
| 06-27-2020 | 23061 |
| 06-28-2020 | 20482 |
| 06-29-2020 | 24263 |
| 06-30-2020 | 19452 |
