# Supplementary material for: Evolving Face Mask Guidance During a Pandemic and Potential Harm to Public Perception: Infodemiology Study of Sentiment and Emotion on Twitter
Source: J Med Internet Res. 2023 Feb 27;25:e40706. doi: 10.2196/40706 (PMC9973548; doi:10.2196/40706)
Supplement: Multimedia Appendix 2 [file jmir_v25i1e40706_app2.docx]

**Table S2.** Number of tweets for a given day between April 1, 2021, to June 13, 2021

| Date | Number of Tweets |
| --- | --- |
| 03-31-2021 | 1087 |
| 04-01-2021 | 5163 |
| 04-02-2021 | 4540 |
| 04-03-2021 | 3319 |
| 04-04-2021 | 2822 |
| 04-05-2021 | 4959 |
| 04-06-2021 | 5655 |
| 04-07-2021 | 4523 |
| 04-08-2021 | 4192 |
| 04-09-2021 | 4231 |
| 04-10-2021 | 4378 |
| 04-11-2021 | 3941 |
| 04-12-2021 | 4007 |
| 04-13-2021 | 3673 |
| 04-14-2021 | 3893 |
| 04-15-2021 | 4748 |
| 04-16-2021 | 4403 |
| 04-17-2021 | 3683 |
| 04-18-2021 | 4467 |
| 04-19-2021 | 5800 |
| 04-20-2021 | 4842 |
| 04-21-2021 | 3435 |
| 04-22-2021 | 4945 |
| 04-23-2021 | 4319 |
| 04-24-2021 | 3654 |
| 04-25-2021 | 4185 |
| 04-26-2021 | 5527 |
| 04-27-2021 | 10502 |
| 04-28-2021 | 6428 |
| 04-29-2021 | 4062 |
| 04-30-2021 | 3784 |
| 05-01-2021 | 2970 |
| 05-02-2021 | 2964 |
| 05-03-2021 | 4761 |
| 05-04-2021 | 4905 |
| 05-05-2021 | 3934 |
| 05-06-2021 | 3429 |
| 05-07-2021 | 3277 |
| 05-08-2021 | 2557 |
| 05-09-2021 | 2624 |
| 05-10-2021 | 3782 |
| 05-11-2021 | 3717 |
| 05-12-2021 | 3844 |
| 05-13-2021 | 18754 |
| 05-14-2021 | 15966 |
| 05-15-2021 | 7534 |
| 05-16-2021 | 5766 |
| 05-17-2021 | 7989 |
| 05-18-2021 | 7196 |
| 05-19-2021 | 5628 |
| 05-20-2021 | 4606 |
| 05-21-2021 | 4288 |
| 05-22-2021 | 2887 |
| 05-23-2021 | 2957 |
| 05-24-2021 | 3734 |
| 05-25-2021 | 3276 |
| 05-26-2021 | 2801 |
| 05-27-2021 | 3158 |
| 05-28-2021 | 3254 |
| 05-29-2021 | 2528 |
| 05-30-2021 | 2313 |
| 05-31-2021 | 1956 |
| 06-01-2021 | 2698 |
| 06-02-2021 | 3826 |
| 06-03-2021 | 3406 |
| 06-04-2021 | 2893 |
| 06-05-2021 | 2262 |
| 06-06-2021 | 2009 |
| 06-07-2021 | 2513 |
| 06-08-2021 | 2313 |
| 06-09-2021 | 2770 |
| 06-10-2021 | 2449 |
| 06-11-2021 | 2266 |
| 06-12-2021 | 1739 |
| 06-13-2021 | 1453 |
